# Hysteroscopy in the Treatment of Myometrial Scar Defect (Diverticulum) Following Cesarean Section Delivery: A Systematic Review and Meta-Analysis

**DOI:** 10.7759/cureus.11317

**Published:** 2020-11-03

**Authors:** Bdour H Al Mutairi, Ibtehal Alrumaih

**Affiliations:** 1 Obstetrics and Gynecology, Maternity and Children Hospital, Qassim, SAU

**Keywords:** hysteroscopy, diverticulum, niche, isthmocele, caesarian scar

## Abstract

Various management approaches have been developed to treat symptoms and prevent complications of the cesarean diverticulum. This systematic review aims to report the outcomes and fertility-related effects of hysteroscopy on women with myometrial scar defects after the cesarean section. Following the formulation of the patient/population, intervention, comparison, and outcomes (PICO) criteria, a systematic search was conducted on seven databases. Finally, a total of 18 studies were included for this systematic review and meta-analysis. All of the included patients suffered from post-cesarean section scars and presented with abnormal bleeding, pain, or secondary infertility. The overall pooled symptomatic improvement rate was 78.83% (95% CI: 72.46-85.76%); however, there was significant heterogeneity among the analyzed studies (I^2^=87%; p-value: <0.001) and a significant risk of bias (p-value: <0.001). The overall resolution/improvement rate after adjusting for possible bias was higher, 92.82% (95% CI: 85.17-100%). The overall pregnancy rate was 69.77% (95% CI: 59.03-82.48%), while in the individual studies the rates varied, ranging from 25% to 80%. Nevertheless, there was moderate heterogeneity among the included studies (I^2^=56%; p-value=0.011). In contrast, there was no significant risk of bias among the included studies (p-value=0.100). Furthermore, the meta-regression analyses did not show any significant effect of different follow-up durations on the overall effect size for both outcomes. In conclusion, there is still a need for high-quality, comparative studies with larger sample sizes and long-term follow-up periods to draw firm conclusions. Moreover, future studies should consider the minimum myometrial thickness that is sufficient to complete a healthy pregnancy.

## Introduction and background

Delivery through cesarean sections has been on the rise recently [1]. Despite being less painful than a natural delivery, it is associated with many complications. A cesarean scar may be complicated by rupture or dehiscence, uterine rupture, and abnormally adherent placenta, and hence may affect pregnancy [2]. During the ultrasonography examination, a triangular section is often noticed within the myometrium in the lower uterine segment where the cesarean section was performed [3,4]. It has been reported in a large portion of pregnant women after routine evaluations with transvaginal sonography and transvaginal sonohysterography [5-7]. It has been observed in women after cesarian operations as an anechoic “filling defect” area, most probably occurring after multiple cesarean deliveries. This defect is also known as the niche and was first reported in pregnant women in 2001 [3,5,6], and its definition is still controversial [8]. The two parts that form it are as follows: a hypoechoic part representing the defect, and a scar fibrotic tissue pouch within the myometrium.

The most common symptom of the niche is postmenstrual spotting or prolonged menstruation, which is usually associated with discomfort and is an alarm signal for gynecology examination [3,5]. This was first reported in 1975, which was followed by many studies later on [4,6,9-10]. It occurs most probably due to the retention of blood in the niche as it can hinder the accumulated blood after pregnancy [4,11,12]. Another explanation is the formation of fragile blood vessels that can easily bleed and cause pain [13]. The prevalence rate of postmenstrual spotting is 30% in pregnant women with cesarean diverticula [7]. Other symptoms such as dysmenorrhea, hypogastric pain, micturition disorders, secondary infertility, and other chronic conditions have also been reported [7].

Various management approaches have been developed to treat symptoms and prevent complications of the cesarean diverticulum. Surgical removal of the uterus (hysterectomy) has been effectively used as a radical treatment method; however, it leads to infertility, and hence other approaches have been developed to help women maintain their fertility [4,14,15]. These include drainage of the accumulated blood in the uterine wall and coagulation of the fragile vessels to prevent further blood production (hysteroscopic resection) [16-20], hormonal intervention to decrease menstrual periods, and blood or abdominal or vaginal repairing of the niche by laparoscopy, or laparotomy [21-24]. The risks associated with invasive procedures include dehiscence and uterine bleeding or rupture [21]. Moreover, they have not been investigated by an adequate number of studies and, therefore, should be performed only when other non-invasive approaches are not available or have failed. 

The most recent meta-analysis describing hysteroscopy in the treatment of myometrial scar was limited by many shortcomings [25], as follows: the search was confined to publications until 2018, non-inclusion of some studies before that date, no assessment or adjustment for risk of bias, no exploration for heterogeneity sources, no control for differences in follow-up duration, no sensitivity analysis, and the inclusion of only a small number of studies. These drawbacks resulted in “very low” evidence, as per their own expression. Keeping that in mind, we aim to provide a higher quality of evidence by including the recently published reports that describe hysteroscopy in the treatment of myometrial scar defects after cesarean section deliveries.

## Review

Search strategy and study selection

According to the accepted methodology, a Preferred Reporting Items for Systematic Reviews and Meta-Analyses (PRISMA) checklist for systematic review and meta-analysis was formulated using the patient/population, intervention, comparison, and outcomes (PICO) criteria for this study [26]. The PICO criteria were adapted for our purpose as follows: population: symptomatic and/or infertile women with cesarean scar defects; intervention: isthmocele treated with hysteroscopy; control: untreated isthmocele or that treated non-surgically; outcomes: improvement and fertility outcomes following hysteroscopy. Symptomatic improvement was defined as the improvement or complete resolution of the symptoms, including abnormal uterine bleeding (AUB) or pelvic pain, following hysteroscopic treatment. Meanwhile, the pregnancy rate was defined as the number of women aiming to get pregnant and conceived successfully following the hysteroscopic treatment. A search strategy was developed using the appropriate keywords for collecting the relevant studies: (Hysteroscopy OR Hysteroscopic) AND (Cesarean OR C-Section) AND (Scar OR Diverticulum OR Isthmocele OR Cesarean Scar OR Niche) AND (Improvement OR Resolution OR Fertility OR Pregnancy Rate OR Gestation). The search was conducted on seven databases on June 19, 2020, including PubMed, Google Scholar, System for Information on Grey Literature in Europe (SIGLE), Scopus, Web of Science [Institute for Scientific Information (ISI)], Virtual Health Library (VHL), Cochrane Database, and The New York Academy of Medicine (NYAM). The inclusion criteria were as follows: studies involving women undergoing hysteroscopy for cesarean scar regardless of patient age, language, or study design. Editorials, case series studies involving less than 10 patients, and review articles were excluded from the study. After the transmission of the systematic search results, two reviewers conducted the systematic search and screened the titles and abstracts independently, which was followed by a full-text screening for selecting the relevant studies. In both steps, the results were further checked by a third reviewer and a senior author if necessary.

Data extraction

An extraction sheet was developed based on the pilot extraction of at least three included papers. The extraction sheet included patients’ characteristics, outcomes, and risk of bias tool. Data extraction was done by the authors and rechecked by a librarian for weeding out wrong or inappropriate data.

Quality assessment

Based on the heterogeneity of the included study designs, a decision to use the National Institutes of Health (NIH) quality assessment tool for rating the quality of evidence was proposed [27] (see Appendix for details). The quality of each study was determined by the authors and a librarian from the Ministry of Health (see Appendix for details).

Statistical analysis

All data were analyzed using the R software, version 4.0.1 [28]. Using the “meta” package, the prevalence rates of different outcomes were calculated [29]. The corresponding 95% CIs of the pooled effect size were calculated using the random effects model due to the presence of heterogeneity. Heterogeneity was assessed with Q statistics and I^2^ test, and an I^2^ value of >50% or a p-value of <0.05 was considered statistically significant [30].

The publication bias was assessed using Egger’s regression test [31] and represented graphically by Begg’s funnel plot [32] when there were 10 or more studies. A p-value of <0.10 in Egger’s regression test was considered significant. Whenever publication bias was found, the trim-and-fill method of Duvall and Tweedie was applied to add studies that appeared to be missing [33] to enhance the symmetry. A leave-one-out sensitivity analysis was also performed by iteratively removing one study at a time to confirm that the study findings were not driven by any single study.

Search results

**Figure 1:**
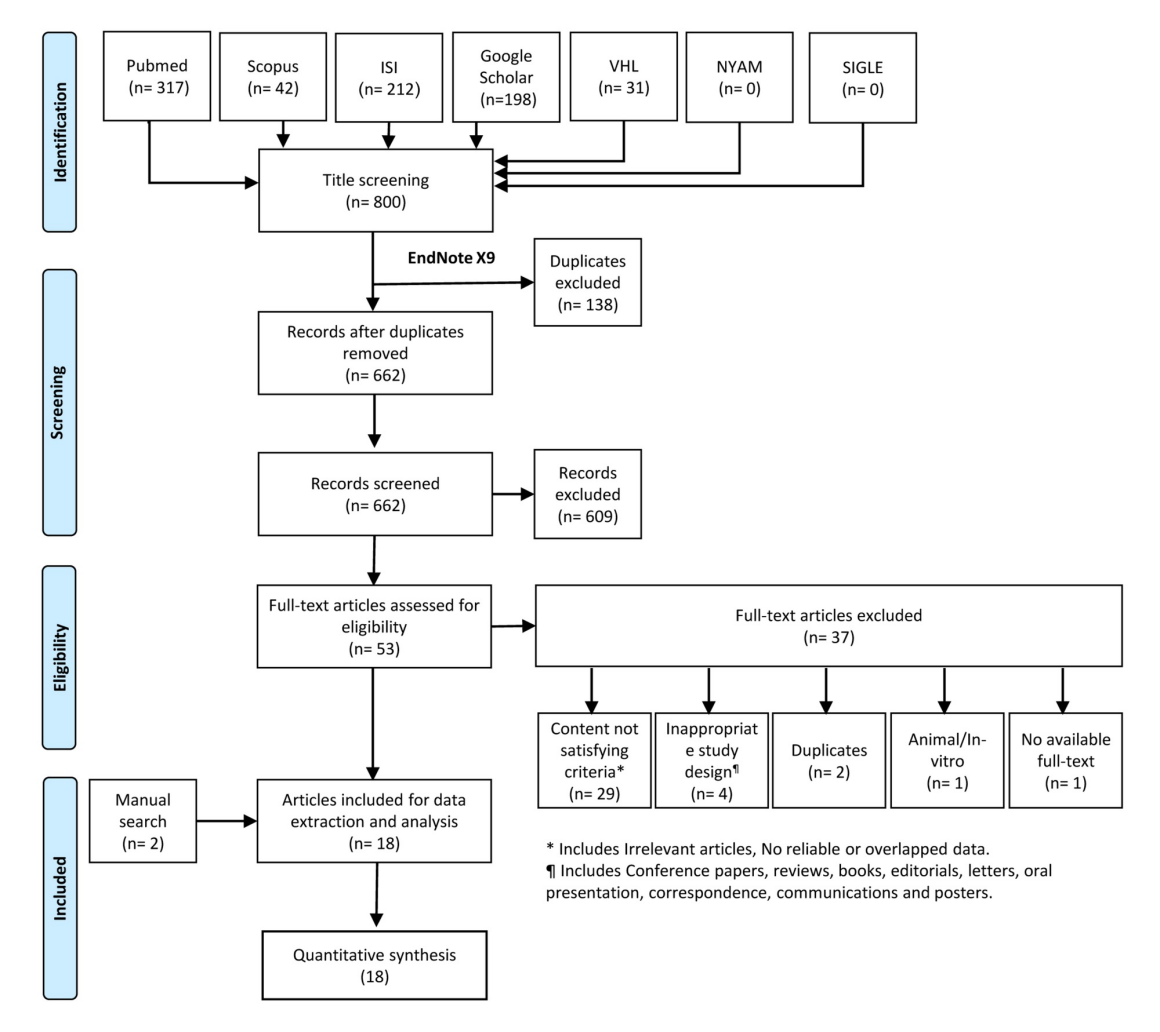
PRISMA flowchart of the search and screening process PRISMA: Preferred Reporting Items for Systematic Reviews and Meta-Analyses; ISI: Institute for Scientific Information; VHL: Virtual Health Library; NYAM: New York Academy of Medicine; SIGLE: System for Information on Grey Literature in Europe

A total of 800 records was found after searching within seven databases. The exclusion of 138 records was done after being determined as duplicates by the EndNote software (Clarivate, Philadelphia, PA). Furthermore, 609 records were excluded after the title and abstract screening. Further full-text screening resulted in the inclusion of 16 papers and another two papers were added after performing a manual search. In total, 18 studies were included for this systematic review and meta-analysis (Figure 1).

Study characteristics and the quality of the included studies

A total of 18 studies were included with a total sample size of 1,165 individuals, and the individual sample sizes ranged from 18 to 273. The mean age of the included population ranged from 27.8 to 40 years (Table 1). Regarding the risk of bias, four studies were graded as good quality, 12 were graded as fair quality, and two studies were of poor quality (Tables 2-5 in the Appendix section).

**Table 1:** Characteristics of the included studies AUB: abnormal uterine bleeding; CSD: cesarean section diverticula; SD: standard deviation; #: median; NIH: National Institutes of Health

Author/year/country	Study design	Sample size	Age (years)	Objective	Main conclusions	Quality assessment tool	Overall quality
			Mean ±SD				
Fabres et al./2005/Chile [16]	Retrospective cohort	24	36	To assess the effectiveness of hysteroscopic surgery to correct the anatomic defect and eliminate the bleeding disturbance in a group of women with this symptom	Previous cesarean delivery scar defect may be the cause of intermenstrual bleeding, and it is possible that it may also impair fertility, but it can be successfully treated by hysteroscopic surgery	NIH Quality Assessment of Controlled Intervention Studies	Good
			Range:29–41				
Wang et al./2010/Taiwan [19]	Retrospective cohort	57	38.8 ±5.8 (improving) and 36.3 ±5.1 (stationary)	To evaluate the efficacy of resectoscopic remodeling of the cesarean section scar in the management of post-cesarean section AUB	Resectoscopic uterine remodeling is an appropriate therapy in patients with post-cesarean section AUB and an anteflexed uterus	NIH Quality Assessment of Controlled Intervention Studies	Fair
Gubbini et al./2008/Italy [20]	Prospective cohort	26	Range:29–42	To assess the effectiveness of a hysteroscopic surgical technique to correct the anatomic defect and thereby eliminate the symptoms	The “isthmocele” represents a possible consequence of one or more cesarean deliveries and may be symptomatic in some women. It is a defect that can be easily diagnosed by hysteroscopy and successfully treated by resectoscopic technique	NIH Quality Assessment of Controlled Intervention Studies	
Shi et al./2020/China [34]	Retrospective cohort	124	35.0 ± 5.0	To describe the improvement after hysteroscopic resection of CSD in women without childbearing intention, and to explore the variables associated with poor prognosis	A hysteroscopic repair might be an appropriate method for CSD in women who have no childbearing intentions. The timing of surgery and the number of cesarean sections seem to be factors influencing the postoperative improvement of cesarean section defects	NIH Quality Assessment of Controlled Intervention Studies	Good
Calzolari et al./2019/Spain [35]	Retrospective cohort	35	Range: 33–38	To evaluate the prevalence of infertility among patients with isthmocele, the resolution of symptoms, and infertility outcomes after hysteroscopic isthmoplasty	Definition of a subgroup of patients at higher risk of being infertile after the diagnosis of isthmocele and a subgroup of patients who could benefit the most in terms of fertility after minimally invasive hysteroscopic surgery	NIH Quality Assessment of Controlled Intervention Studies	Fair
Albornoz et al./2017/Spain [36]	Prospective case series	38	40	To assess the effectiveness of hysteroscopic surgical treatment of isthmocele in women with associated symptoms such as pelvic pain and AUB	Hysteroscopic correction of symptomatic isthmoceles may constitute a safe and effective technique for patients who present with AUB and pelvic pain	NIH Quality Assessment Tool for Case Series Studies	Fair
			Range: 31–47				
Feng et al./2012/China [37]	Retrospective cohort	62	34 ±5.4	To estimate the usefulness of hysteroscopy in the diagnosis and treatment of postcesarean scar defect	Hysteroscopy is an accurate means of diagnosis apart from surgical correction	NIH Quality Assessment of Controlled Intervention Studies	Good
Abdou and Ammar/2018/Egypt [38]	Randomized non-blinded trial	56	27.79 ±3.52	To evaluate the role of hysteroscopic repair of cesarean scar defect in patients with secondary infertility	Hysteroscopic repair of cesarean scar defect in women with secondary infertility and a residual myometrial thickness of 3 mm offers a minimally invasive approach with a high success rate and no complications	NIH Quality Assessment of Controlled Intervention Studies	Poor
Cohen et al./2020/Israel [39]	Retrospective cohort	39	37.2	To evaluate the fertility outcomes of symptomatic patients following hysteroscopic niche resection	Hysteroscopic niche resection should be considered an effective treatment in patients suffering from secondary infertility	NIH Quality Assessment of Controlled Intervention Studies	Fair
			Range: 34–41				
Shapira et al./2019/Israel [40]	Retrospective cohort	67	38 ±5.5	To evaluate the efficacy of extensive hysteroscopic cesarean scar niche excision in symptomatic patients	Extensive hysteroscopic surgical excision of the cesarean scar niche should be considered in symptomatic patients suffering from irregular menstrual bleeding. The quality of the excision at the apex of the niche could be associated with a higher success rate	NIH Quality Assessment of Controlled Intervention Studies	Fair
Xie et al./2014/China [41]	Retrospective cohort	77	33.26 ±3.78	To compare the efficacy of vaginal surgery and operative hysteroscopy for the treatment of cesarean-induced isthmocele	The therapeutic efficacy of vaginal surgery is superior to operative hysteroscopy in the treatment of cesarean-induced isthmocele	NIH Quality Assessment of Controlled Intervention Studies	Fair
Tsuji et al./2017/Japan [42]	Prospective cohort	18	Range: 31–39	To assess the impact of hysteroscopic surgery on isthmocele associated with cesarean section scar	Hysteroscopic surgery is effective in increasing the residual myometrial thickness and reducing the size of isthmocele	NIH Quality Assessment of Controlled Intervention Studies	Fair
Tanimura et al./2015/Japan [43]	Prospective cohort	22	37#	To assess the efficacy of endoscopic repair for secondary infertility caused by post-cesarean scar defect	Infertility associated with post-cesarean scar defect, cesarean scar syndrome, is caused by the retention of bloody fluid in the uterine cavity and scarring. Endoscopic treatment, such as hysteroscopy or laparoscopy, was effective for cesarean scar syndrome	NIH Quality Assessment of Controlled Intervention Studies	Fair
			Range:32–43				
Li et al./2014/China [44]	Retrospective cohort	41	34.8 ± 4.0	To examine the treatment of previous cesarean delivery scar defect after cesarean delivery and the feasibility of laparoscopic uterine repair or hysteroscopic scar excision	Women with a history of cesarean delivery combined with irregular perimenstrual bleeding should undergo combined hysteroscopy and ultrasound examination to detect latent scar defects. In diagnosed cases, in those who desired future pregnancies and had a residual myometrial thickness of <3.5 mm or a defect that accounted for ≥50% of the anterior uterine wall, laparoscopic surgical repair was performed with good postoperative anatomic outcomes	NIH Quality Assessment of Controlled Intervention Studies	Poor
Florio et al./2011/Italy [45]	Retrospective case-control study	39	35 ±4.1	To compare the effectiveness of hysteroscopic correction and hormonal treatment to improve symptoms (postmenstrual AUB, pelvic pain localized in the suprapubic site) associated with isthmocele	Resectoscopic surgery is a valid way to treat patients with symptoms of prolonged postmenstrual uterine bleeding caused by isthmocele. Data from this study also indicate that resectoscopy may be the first choice because it is minimally invasive and yields good therapeutic results	NIH Quality Assessment of Case-Control Studies	Fair
Muzii et al./2017/Italy [46]	Prospective case-control study	47	39.55 ±4.55	To assess the feasibility and efficacy of surgical hysteroscopic treatment of cesarean-induced isthmocele on symptom relief	This is the first prospective controlled trial demonstrating better outcomes of resectoscopic treatment of isthmocele in solving symptoms compared with expectant management	NIH Quality Assessment of Case-Control Studies	Fair
Perez-Medina et al./2013/Spain [47]	Retrospective cohort	273	32.2#	To describe the feasibility of office hysteroscopy in patients with pregnancy-related problems such as retained trophoblastic tissue, persistent molar tissue, pregnancy with in situ intrauterine devices, isthmocele, embryoscopy, and osseous metaplasia	Office hysteroscopy is a safe and minimally invasive treatment for pregnancy-related conditions, with good clinical and functional results	NIH Quality Assessment of Controlled Intervention Studies	Good
			Range:15–41				
Raimondo et al./2020/Italy [48]	Prospective cohort	120	39.2 ±4.5	To evaluate prospectively in 120 consecutive isthmocele patients	Surgical treatment of cesarean-induced isthmocele by operative hysteroscopy may represent the best choice in symptomatic women because of its minimal invasiveness and beneficial therapeutic results	NIH Quality Assessment of Controlled Intervention Studies	Fair

Symptomatic improvement

**Figure 2:**
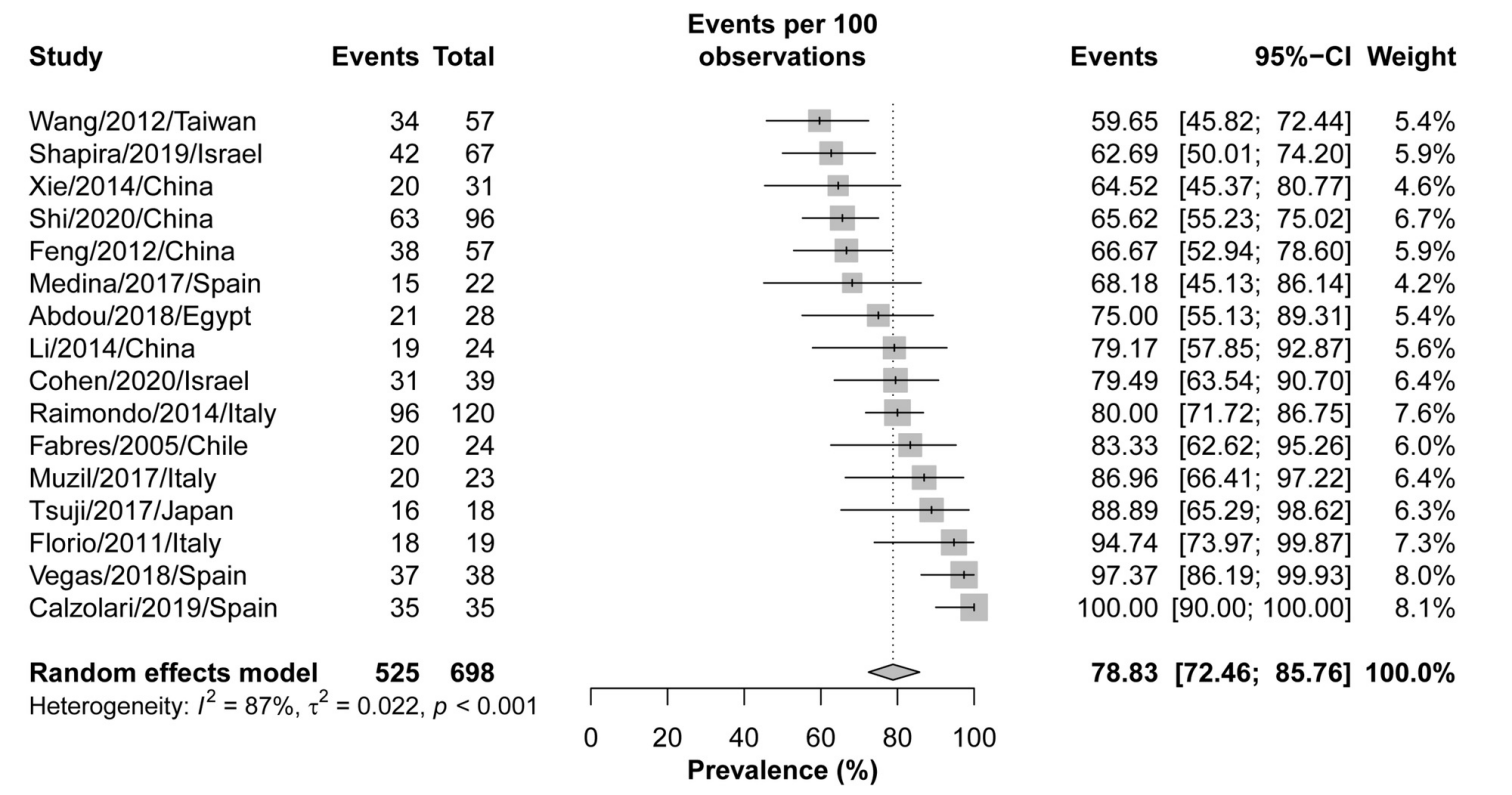
Forest plot for symptomatic improvement following hysteroscopic treatment of myometrial scar defect (diverticulum)

**Figure 3:**
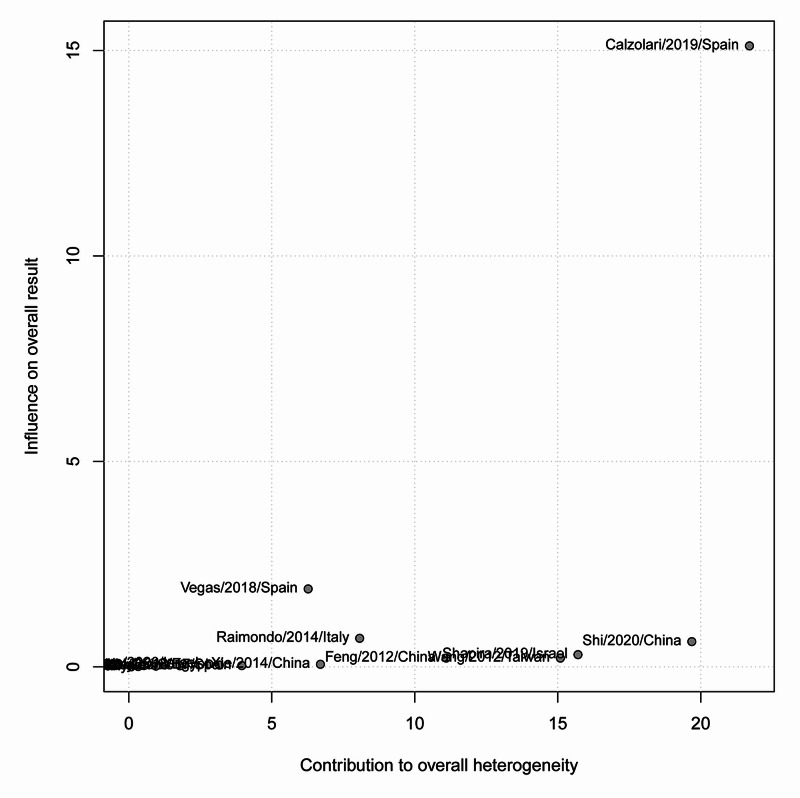
Baujat plot of the contribution of each study to the overall heterogeneity

**Figure 4:**
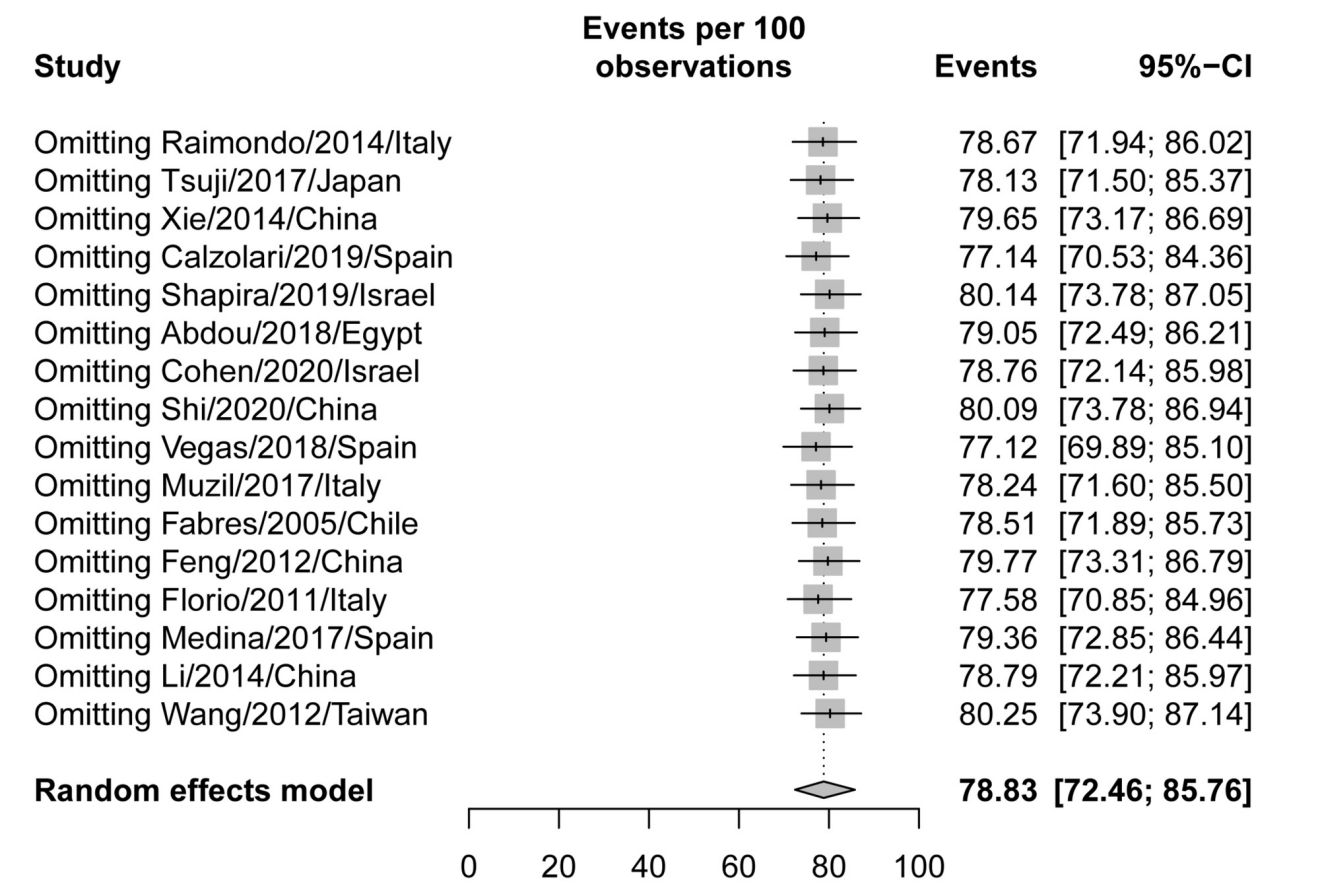
A leave-one-out sensitivity analysis of the symptomatic improvement outcomes

Symptomatic improvement was defined as the improvement or complete resolution of the symptoms, including AUB or pelvic pain, following hysteroscopic treatment. A total of 18 studies, with 698 patients, were included in the analysis of the last follow-up assessment of symptoms resolution/improvement following hysteroscopic treatment. The overall pooled improvement rate was 78.83% (95% CI: 72.46-85.76%); however, there was significant heterogeneity among the analyzed studies (I^2^=87%; p-value: <0.001) (Figure 2). The contribution of each study to the overall heterogeneity is shown in Figure 3. The studies by Shi et al. [34] and Calzolari et al. [35] alone contributed >40% of the overall estimated heterogeneity. The leave-one-out sensitivity analysis showed the same high rates of improvement/resolution, indicating that the effects are not driven by one study (Figure 4).

**Figure 5:**
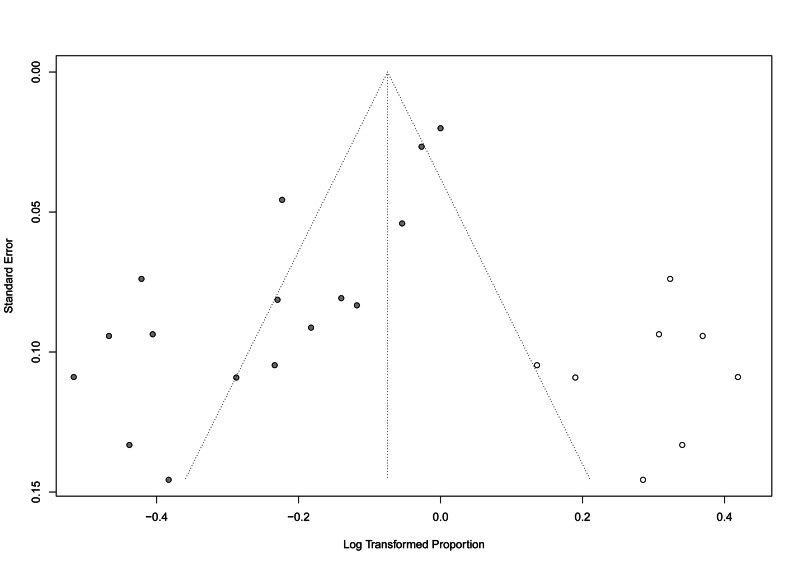
Funnel plot of the symptomatic improvement outcomes* *Five studies were added on the right side to enhance symmetry

**Figure 6:**
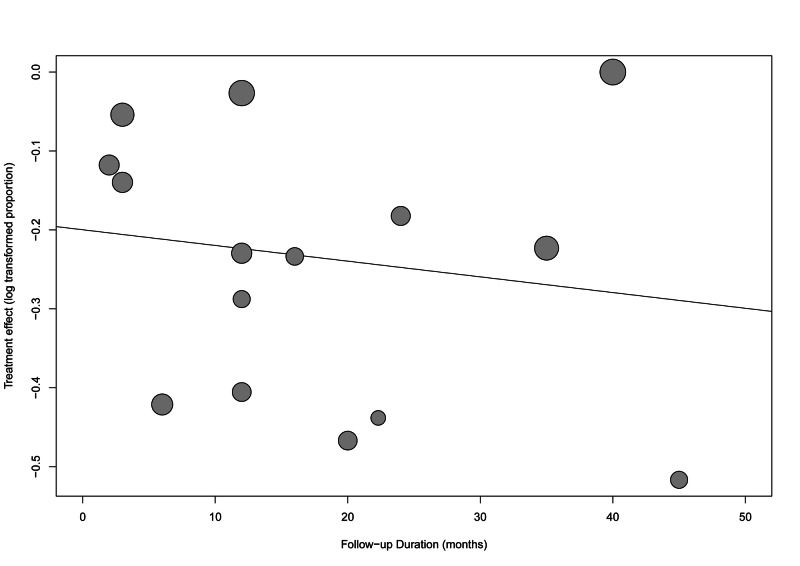
Meta-regression of the follow-up duration and its effect on the symptomatic improvement outcomes

There was a significant risk of bias (p-value: <0.001) when tested using Egger’s regression test. The funnel plot with the trim-and-fill method added eight studies to adjust for funnel plot symmetry, as shown in Figure 5. The overall resolution/improvement rate after adjusting for possible bias was higher, 92.82% (95% CI: 85.17-100%). The meta-regression analysis did not show any significant effect of different follow-up durations on the overall effect size (estimate=0.002, standard error=0.004, p-value=0.588) (Figure 6).

Pregnancy rate

**Figure 7:**
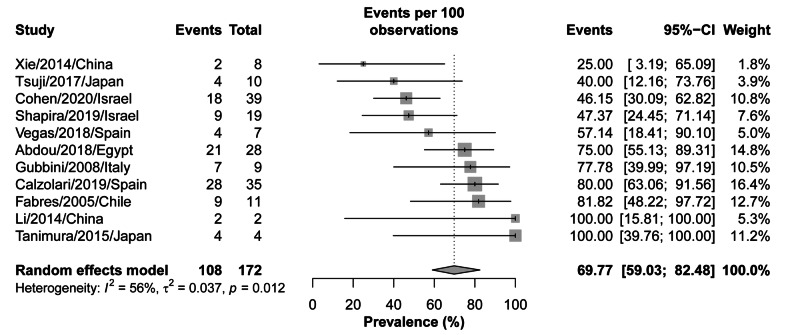
Forest plot for pregnancy rates following hysteroscopic treatment of myometrial scar defect (diverticulum)

The pregnancy rate was defined as the number of women who aimed to get pregnant and conceived successfully following the hysteroscopic treatment. The data of pregnancy rates following hysteroscopic treatment, at the last follow-up time point, were available for 11 studies, including 172 patients with secondary infertility. The overall pregnancy rate was 58.71% (95% CI: 59.03-82.48%), while in the individual studies the rates varied, ranging from 25% to 100%. Nevertheless, there was moderate heterogeneity among the included studies (I^2^=56%; p=0.012) (Figure 7).

**Figure 8:**
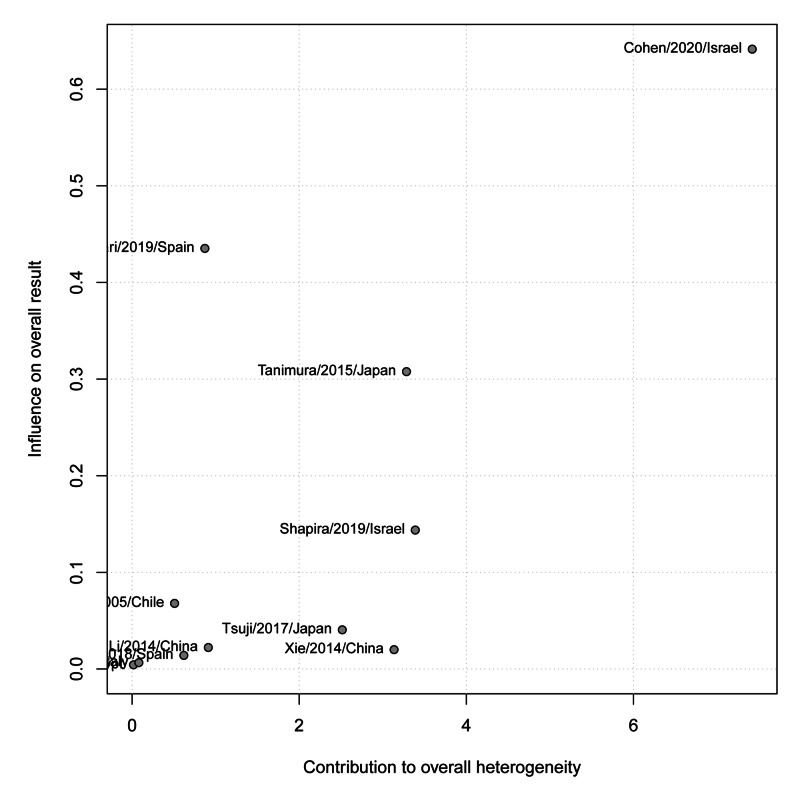
Baujat plot of the contribution of each study to the overall heterogeneity (pregnancy rates)

**Figure 9:**
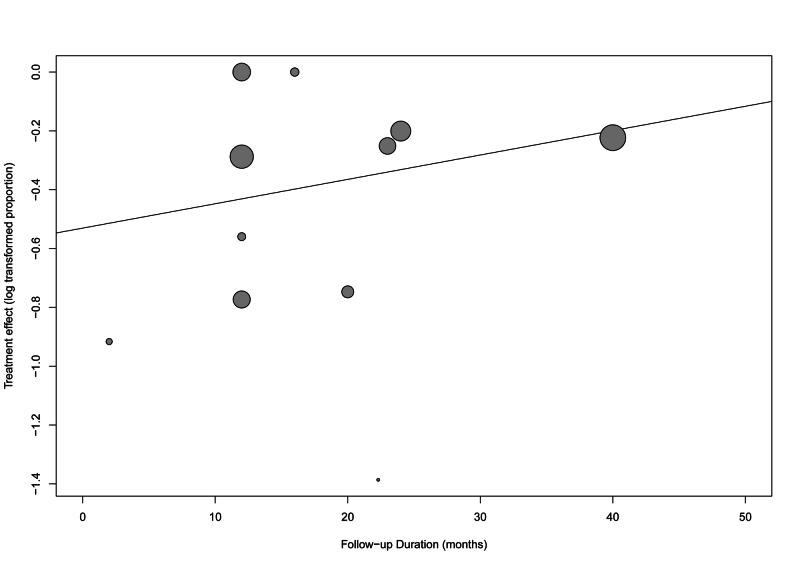
Meta-regression of the follow-up duration and its effect on pregnancy rate outcomes

The contribution of each study to the overall heterogeneity is shown in Figure 8. In contrast, there was no significant risk of bias among the included studies (p-value=0.100). Moreover, the meta-regression analysis did not show any significant effect of different follow-up durations on the overall effect size (estimate=0.008, standard error=0.009, p-value=0.360) (Figure 9).

Discussion

Post-cesarean diverticula complications vary among pregnant women as their dimensions change throughout pregnancy. Since they affect a sensitive part of women’s bodies and their management is essential pertaining to the prognosis of their sexual lives, niches ought to be treated carefully. Management approaches include non-invasive therapies, which consist of hormonal treatments to decrease the time or blood flow of the menstrual cycles, and surgical therapies, which aim to mechanically stop the bleeding from the target area. One of the most minimally invasive and effective procedures is hysteroscopy. It is widely used to inspect the uterus and help in the diagnosis of abnormal vaginal bleeding. Moreover, it is associated with fewer morbidities and allows patients to continue with their pregnancies safely in a short time. As it is a minimally invasive procedure for managing AUB, it has been associated with shorter times of bleeding and high levels of patient satisfaction after the procedure.

All of the included patients suffered from post-cesarean section scars and presented with abnormal bleeding, pain, or secondary infertility. This is an updated meta-analysis on the effect of hysteroscopy in the management of AUB due to post-cesarean scars. This study analysis showed that among 698 patients with post-cesarean complications including bleeding, pain, and secondary infertility and who underwent hysteroscopy, the non-adjusted overall improvement was 78.83%. Although the improvement rate was high, the calculated heterogeneity between the included studies was recorded to be significant (p-value: <0.001). However, the heterogeneity in Shi et al. [34] and Calzolari et al. [35] alone contributed to 50% of the overall estimated heterogeneity. This is because of the short period of follow-up, the nature of data selection, and the small sample size of included patients in those studies. Moreover, although Vegas et al. [36] had a 97.37% improvement rate, the rate was not solely influenced by these high results, as the leave-one-out sensitivity analysis showed (Figure 4). A statistical significance was also found in the risk of bias (p-value=0.001). Consequently, there was an adjustment of the asymmetry in the funnel plot representing all studies, and the overall improvement rate was found to be 92.82%. On the other hand, Feng et al. [37] reported a rate of 87% decrease in AUB after management with hysteroscopy, but a higher rate of 100% with laparoscopy, and 93% with the vaginal repair. Moreover, the same author reported the rate of pain relief to be 97% with hysteroscopy and 100% with laparoscopy while secondary infertility decreased in more patients after hysteroscopy [37].

The overall pregnancy rate was found to be 69.77% with a significant heterogeneity (p-value=0.012) among the 11 studies that reported it at the end of the overall follow-up period [35,36,38-42]. Along with the highest improvement rates, Tanimura et al. [43] and Lei et al. [44] also reported the highest pregnancy rates (100%) in women who underwent hysteroscopy as a surgical intervention for niches, while Xie et al. [41] reported the lowest rates (25%). Other endoscopic procedures have also been reported. Many studies reported a significant improvement in the pregnancy rate after performing laparoscopy in patients presenting with secondary infertility [45-47]. Tanimura et al. [43] compared the pregnancy rates between women with niches who underwent hysteroscopy and others who underwent laparoscopy and found that the pregnancy rate was higher in the hysteroscopy group (100%) than the laparoscopy one (55.56%). Combined hysteroscopy-guided laparoscopic arthroplasty was also reported to reflect a high pregnancy rate (80%) [45].

The heterogeneity between the improvement and post-treatment pregnancy rates could be attributed to the difference in methodology and management criteria due to the lack of globally specific definitions. A commonly reported factor for an increased incidence of pregnancy is myometrial thickening. Sufficient thickness will prevent uterine rupture during normal contractions. However, the reported minimal thickness of the myometrium sufficient for safe vaginal births has varied. Lastly, Bujold et al. [49] reported a 2.8-mm minimal thickness in women with a history of post-cesarean diverticula, while Sen et al. reported a lower value of 2.5 [50]. Therefore, vaginal birth should not be performed in patients with myometrial thickness beneath these values, according to these authors [49,50].

To date, this is the largest and most recent systematic review and meta-analysis conducted on this topic. It provides high-quality evidence with a comprehensive assessment of risk-of-bias heterogeneity, heterogeneity, and possible confounding effects of different follow-up durations. Limitations to this study include the considerable heterogeneity in symptomatic improvement outcomes and the lack of defined criteria for hysteroscopic procedures, which indicates that interpretation of the results should be done with caution. However, as mentioned above, no clear criteria have been approved universally. Moreover, the small sample size and the nature of data collection of some of the included studies may have played a role in the risk of bias. Finally, long follow-up periods should be applied to study the long-term outcomes of pregnancy. In this study, however, no significant effect was found regarding the different reported follow-up periods on this study rates (p-value=0.374).

## Conclusions

There is still a need for high-quality, comparative studies with larger sample sizes and long-term follow-up periods to arrive at firm conclusions regarding this topic. Moreover, future studies should consider the minimum myometrial thickness that is sufficient to complete a healthy pregnancy.

## Appendices

**Table 2:** Quality rating of the cohort and cross-sectional studies 1. Was the research question or objective in this paper clearly stated? 2. Was the study population clearly specified and defined? 3. Was the participation rate of eligible persons at least 50%? 4. Were all the subjects selected or recruited from the same or similar populations (including the same time period)? Were inclusion and exclusion criteria for being in the study prespecified and applied uniformly to all participants? 5. Was a sample size justification, power description, or variance and effect estimates provided? 6. For the analyses in this paper, were the exposure(s) of interest measured prior to the outcome(s) being measured? 7. Was the timeframe sufficient so that one could reasonably expect to see an association between exposure and outcome if it existed? 8. For exposures that can vary in amount or level, did the study examine different levels of the exposure as related to the outcome (e.g., categories of exposure, or exposure measured as a continuous variable)? 9. Were the exposure measures (independent variables) clearly defined, valid, reliable, and implemented consistently across all study participants? 10. Was the exposure(s) assessed more than once over time? 11. Were the outcome measures (dependent variables) clearly defined, valid, reliable, and implemented consistently across all study participants? 12. Were the outcome assessors blinded to the exposure status of participants? 13. Was the loss to follow-up after baseline 20% or less? 14. Were key potential confounding variables measured and adjusted statistically for their impact on the relationship between exposure(s) and outcome(s)?

Reference ID	Q1	Q2	Q3	Q4	Q5	Q6	Q7	Q8	Q9	Q10	Q11	Q12	Q13	Q14	Overall quality
Cohen et al./2020/Israel	1	1	1	1	0	1	1	0	1	1	1	0	0	0	Fair
Shi et al./2020/China	1	1	1	1	0	1	1	1	1	1	1	0	1	1	Good
Calzolari et al./2019/Spain	1	1	1	1	0	1	1	1	1	1	1	0	0	0	Fair
Raimondo et al./2020/Italy	1	1	1	1	0	1	1	1	1	1	1	0	0	0	Fair
Shapira et al./2019/Israel	1	1	1	1	0	1	1	1	1	1	1	0	0	0	Fair
Tsuji et al./2017/Japan	1	1	1	1	0	1	1	1	1	1	1	0	0	0	Fair
Xie et al./2014/China	1	1	1	1	0	1	1	1	1	1	1	0	0	0	Fair

**Table 3:** Quality rating of the controlled intervention study 1. Was the study described as randomized, a randomized trial, a randomized clinical trial, or an RCT? 2. Was the method of randomization adequate (i.e., use of randomly generated assignment)? 3. Was the treatment allocation concealed (so that assignments could not be predicted)? 4. Were study participants and providers blinded to the treatment-group assignment? 5. Were the people assessing the outcomes blinded to the participants' group assignments? 6. Were the groups similar at baseline on important characteristics that could affect outcomes (e.g., demographics, risk factors, comorbid conditions)? 7. Was the overall drop-out rate from the study at endpoint 20% or lower of the number allocated to treatment? 8. Was the differential drop-out rate (between treatment groups) at endpoint 15 percentage points or lower? 9. Was there high adherence to the intervention protocols for each treatment group? 10. Were other interventions avoided or similar in the groups (e.g., similar background treatments)? 11. Were outcomes assessed using valid and reliable measures and implemented consistently across all study participants? 12. Did the authors report that the sample size was sufficiently large to be able to detect a difference in the main outcome between groups with at least 80% power? 13. Were the outcomes reported or subgroups analyzed prespecified (i.e., identified before analyses were conducted)? 14. Were all randomized participants analyzed in the group to which they were originally assigned, i.e., did they use an intention-to-treat analysis?

Reference ID	Q1	Q2	Q3	Q4	Q5	Q6	Q7	Q8	Q9	Q10	Q11	Q12	Q13	Q14	Overall quality
Abdou and Ammar/2018/Egypt	1	0	0	0	1	0	0	0	1	1	1	0	1	1	Poor

**Table 4:** Quality rating of the case series study 1. Was the study question or objective clearly stated? 2. Was the study population clearly and fully described, including a case definition? 3. Were the cases consecutive? 4. Were the subjects comparable? 5. Was the intervention clearly described? 6. Were the outcome measures clearly defined, valid, reliable, and implemented consistently across all study participants? 7. Was the length of follow-up adequate? 8. Were the statistical methods well-described? 9. Were the results well-described?

Reference ID	Q1	Q2	Q3	Q4	Q5	Q6	Q7	Q8	Q9	Overall quality
Albornoz et al./2017/Spain	1	1	1	0	1	1	0	1	1	Fair

**Table 5:** Quality rating of the case-control study 1. Was the research question or objective in this paper clearly stated and appropriate? 2. Was the study population clearly specified and defined? 3. Did the authors include a sample-size justification? 4. Were controls selected or recruited from the same or similar population that gave rise to the cases (including the same timeframe)? 5. Were the definitions, inclusion and exclusion criteria, algorithms or processes used to identify or select cases and controls valid, reliable, and implemented consistently across all study participants? 6. Were the cases clearly defined and differentiated from controls? 7. If less than 100 percent of eligible cases and/or controls were selected for the study, were the cases and/or controls randomly selected from those eligible? 8. Was there use of concurrent controls? 9. Were the investigators able to confirm that the exposure/risk occurred prior to the development of the condition or event that defined a participant as a case? 10. Were the measures of exposure/risk clearly defined, valid, reliable, and implemented consistently (including the same time period) across all study participants? 11. Were the assessors of exposure/risk blinded to the case or control status of participants? 12. Were key potential confounding variables measured and adjusted statistically in the analyses? If matching was used, did the investigators account for matching during study analysis?

Reference ID	Q1	Q2	Q3	Q4	Q5	Q6	Q7	Q8	Q9	Q10	Q11	Q12	Overall quality
Muzii et al./2017/Italy	1	1	0	1	1	1	0	0	1	1	1	0	Fair
